# An empirical study of entrepreneurial leadership and fear of COVID-19 impact on psychological wellbeing: A mediating effect of job insecurity

**DOI:** 10.1371/journal.pone.0284766

**Published:** 2023-05-12

**Authors:** Tajana Guberina, Ai Min Wang, Bojan Obrenovic

**Affiliations:** 1 School of Management, Wuhan University of Technology, Wuhan, China; 2 Zagreb School of Management, Zagreb, Croatia; 3 Luxembourg School of Business, Luxembourg, Luxembourg; St John’s University, UNITED STATES

## Abstract

The empirical study proposes a model for investigating the effect of entrepreneurial leadership on job insecurity and employee psychological wellbeing during COVID-19 based on the combined theoretical grounds of The Conservation of Resources Theory and Social Learning. To explore the job insecurity relationship with psychological wellbeing, and measure the impact of Fear of COVID-19, an empirical study was conducted on a sample of 408 employees in Croatia. The data of the cross-sectional study was collected in November and December 2020. A strong influence of job insecurity on the psychological wellbeing of employees has been identified. Furthermore, fear of COVID-19 was found to have adverse psychological effects on wellbeing. The theorized positive impact of entrepreneurial leadership on job insecurity was not supported by the evidence. The strong point of our contribution lies in the finding that the entrepreneurial leadership style alone does not buffer against job insecurity, thus pointing that the more comprehensive inquiry into other organizational factors, such as coping, learning abilities, developmental opportunities, personal disposition, and pressure bearing. The research is the first step toward enhancing our understanding of the entrepreneurial dimension of transactional psychology. The observations we recorded have implications for research into the study of the mental processes and their impact on organizational proactive behavior.

## Introduction

The beginning of 2020 saw the outbreak of novel corona virus, which brought about unprecedented consequences for the economy and society alike. It caused a sudden global poverty rate and endangered national and public health. As a result of a threat to the economy, many employees were laid off; others were left fearing their employment [1]. Businesses were forced to shut down, while those whose operations were still effective struggled to avoid firing employees and fending turnover. The current study is conducted to provide the integrative reaction to the problems of the ongoing corona virus pandemic in psychology, society, economy, policy, and management. The effects of corona virus have been studied by estimating mental, emotional, behavioral, and existential aspects of people’s psychology [2]. Moreover, social and psychological health problems are related to unemployment, inflation, debt, increasing poverty, and economic insecurity [3]. The danger can be seen in explosive growth in the reported cases of psychological problems [4].

The outbreak proved to be challenging for both employees and team leaders. The toll the pandemic has taken not only threatened corporate viability but the very psychological wellbeing and job security. While the excessive fear of contagion and job loss undoubtedly give rise to organizational disengagement issues such as absenteeism, inattentiveness and general distress on part of the employees, not many studies have undertaken to examine what type of leadership would yield the best results for reassuring and empowering followers to use the present challenge as an opportunity for personal advancement and professional growth. We found several drawbacks in the existing literature. First, the majority of papers in organizational psychology focused on the issue of driving economic value [2], ensuring corporate sustainability [5], and improving employee psychological wellbeing by reducing distress [6]. However, the key role of enterprising managers in providing opportunities for learning and growth as a means to reduce job insecurity was overlooked. Improving psychological capital, fostering go-getting behavior and maintaining a high level of learning to contribute to sustaining a healthy organizational climate. Nielsen et al. (2018) posit it is vital to look into this topic since leaders have a significant role in the psychological distress levels of employees [7]. Notwithstanding the existing support for the correlation between leadership and employee psychological health [8], Birkeland et al. (2016) point out an evident lack of research on entrepreneurial leadership as a specific style and workers’ mental health during stressful events and adversity [9]. The prevailing argument in prior work is that executives are unsure of how to respond to such a large-scale crisis, however, not many empirically supported recommendations were put forward on the best approaches managers and leaders should assume to reduce fear and insecurity while concurrently fostering the entrepreneurial disposition and increasing employees’ work ethics.

In the context of our study, job insecurity and mental health are given high significance during hardship. We theorize that entrepreneurial leadership might be the key to solving fear-driven workplace problems. Researchers studied the effect of suitable leadership styles on personnel behavior, including psychological wellbeing [8, 9]. While some attention in organizational psychology has been given to leadership [10, 11], Zhang (2016) and Fischer (2017) emphasize there is still a lack of research concerning entrepreneurial leadership style [12, 13]. Entrepreneurial leaders are generally characterized by deliberative calm, the ability to solve problems and make quick decisions under pressure, optimize risks and engage in potentially profitable endeavors under high-stress and tumultuous conditions. Unlike empowering leadership, which centers on nurturing organizational engagement by delegation and encouragement [14–16], entrepreneurial leadership has an additional beneficial facet when faced with a crisis and limited resources, i.e., innovation, developing talent and increasing efficiency by transforming problems into profitable opportunities that bring social and economic value [17, 18]. For this reason, we chose to examine the effectiveness of entrepreneurial leadership style in decreasing job insecurity and mitigating fear of COVID-19. Assuming that insecurity stems from a lack of confidence, ambition and enterprising competence, we theorize the issue of employees’ job insecurity during the outbreak could be resolved by teaching opportunity detection, independent thinking, and assertiveness [19]. Empowerment has a significant effect on leadership and individual performance [20]. However, entrepreneurial leaders do more than empower [21]. Under the proper leadership, insecurity may be transformed from stressful to a dynamic, challenge-oriented state wherein employee is faced with an opportunity to acquire new resources, skills and competencies and generate outputs that are deemed both uncertain and relevant [22, 23]. Raising one’s confidence, improving skills and abilities and establishing a lasting role in ensuring a firm’s success minimizes job insecurity and builds resilience in relation to fear of COVID-19 [24].

The past studies lack empirical evidence on leadership and psychological wellbeing in the COVID-19 pandemic context. Based on the empirical evidence, this research aims to fill the existing gap and will provide recommendations for the practice of managing employee psychological health during adversity. Our results are of great value to psychology and organizational literature as we supplied further evidence on the nature of the relationship between entrepreneurial empowerment, workforce protection, and organizational commitment. Our leading research question concerns the eligibility of entrepreneurial leadership style for promoting confidence, increasing workers’ psychological wellbeing, engagement in innovation and reducing fear. Entrepreneurial leadership stimulates a positive atmosphere building on ambitious, resourceful and innovative capabilities, driving followers’ motivation to impersonate congruent entrepreneurial qualities by improving their competencies. This study is the first step toward enhancing our understanding of the entrepreneurial dimension of transactional psychology. The observations we recorded have implications for research into the study of mental processes and their impact on organizational proactive behavior. We provide the previously overlooked concept of EL with additional insight on how to create a healthy and people-centric organizational climate during market uncertainty and complexity.

## Literature review

### Covid-19 economic and business implications

The COVID-19 pandemic has, over the last two years, caused severe hardship for the global economy and public health [25]. While most emerging markets and developing economies (EDMEs) successfully managed to ward off catastrophic side effects during the Great Recession, even they failed to prepare for enforcing such a large-scale crisis response to the coronavirus challenge imposed globally [26, 27]. The significant blow to the global economy had a detrimental impact on human capital [28]. The economic plunge included mass layoffs that lead to poverty, a decrease in purchasing power, and the inaccessibility of healthcare services [26]. The threat of accumulating debt is palpable, posing risks associated with underinvestment, unemployment, and labor force deficits [29]; policymakers are called upon to act strategically, rapidly, and decisively. Consequently, most states are facing hardships to curb further economic harm [30]. Madhav et al. (2017) pointed out that one of the major causes of this damage is human behavioral changes like fear-induced aversion towards the workplace and social gatherings and the influence of mitigation measures [31].

Forthcoming risks are associated with overcoming the challenges regarding businesses’ lack of finance and capacity to ensure job security, thereby decreasing employee wellbeing and failing to protect from the outcomes of adverse risks of COVID-19 on employees’ mental health [32].

As ensuring psychological wellbeing is of significant concern, health professionals, experts, and academics from various fields and disciplines have invested time, attention, and resources into studying the effect the crisis so far had on psychological health and distress [32, 33]. According to Chiu and Lai (2020) [34], measuring the impact of epidemics and pandemics on mental welfare is crucial. Drapeau (2011) and Payton (2009) found distress to be the cause of lack of interest in previously meaningful activities, increased anxiety, sadness as well as insomnia, which was detected as a consequence of workplace stress by Drapeau (2011) and Marchand (2004) [35–37]. Such type of distress is linked to a range of psycho physiological and behavioral symptoms spread over a certain period [37]. A significant source of strain is financial loss and job insecurity. According to the survey, this was proven true among 800 American workers [38]. Individuals’ distress is the outcome of the issue rather than the problem itself [39].

### Theoretical background and key concepts

#### Theoretical background

*Conservation of resources theory*. The Conservation of Resources Theory (COR) provides a framework for the apprehension of the relevance the acquisition of valuable resources has in emotional life and performance [40]. Similarly, they use existing reserves to buffer from further collateral resource losses [41]. According to COR, resources drive individual appraisals of stressful situations and underlie their coping strategies. When a stressful situation presents itself, some of these assets will be invested into maintaining balance and psychological health. Consequently, draining resources to cope will result in decreased engagement in other areas of one’s life, including the energy necessary to excel at work during a crisis. The COR helps to predict the levels of distress and its outcomes [25]. COR posits that stress may emanate from the inability to achieve common social or organizational objectives in a socially shared context or when faced with a common challenge [42]. The COR relates to entrepreneurial leadership and psychological wellbeing in that it explains how through experience and learning employees can identify their needs and engage in resource acquisition, be it directly or indirectly. Entrepreneurial leaders have the power to impact employee wellbeing through providing opportunities for promotion, autonomy, skill enhancement, and acquiring an expertise [43]. Furthermore, job uncertainty deprives people of key resources, such as a stable environment, financial security, consistency and social support. Under the latent deprivation model, insecure workers feel exhausted, discouraged, dissatisfied and more likely to neglect organizational or personal development [44, 45]. Under adaptive strategies, confronting fear and insecurity entails a significant investment of resources [46]. As expected, job uncertainty increases the occurrence of mood instability, anxiety, depression and panic [47].

*Social learning theory*. Buffering against job insecurity can also be explained on the grounds of Social Learning Theory (SLT) [48]. The Theory considers how environmental and cognitive factors collide to shape human behavior. The SLT stresses the relevance of cognitive repetition and observational learning in molding one’s future conduct [49]. Observation, modeling and imitation of the behaviors, attitudes and emotional reactions occur through attention, retention and reproduction. The SLT explains the formation of complex social behaviors through the mediation of motivation and identification. Bandura’s Theory is now commonly applied in organizational context and management studies [50, 51]. Authors have used the SLT to explain the association between work factors, stress and wellbeing [52]. In our model, under Entrepreneurial Leadership employees gain more confidence in their abilities through observing, experimentation, and learning. Furthermore, experiencing the work environment as supportive brings about self-determination and autonomy. When their environment supports independence, employees are intrinsically motivated, innovative, positively charged, and psychologically well-grounded [53].

#### Entrepreneurial leadership

When companies find themselves in a turbulent situation, they need more research into effective leadership styles conducive to organizational and job sustainability [13]. A rapidly changing business environment requires new ways of building the right capabilities by which business owners and managers can instantly respond to ongoing changes. Remarkably, the importance of entrepreneurial leadership is increasing day by day to quickly adapt to ongoing changes in a global economy. In this regard, it should be mentioned that entrepreneurial leadership is not just one trait but involves the combination of different personality traits. The ability to anticipate, envision, strategic thinking, and teamwork are some of the examples of personality traits that entrepreneurial leaders demonstrate.

The more traditional leadership relied heavily on a rigid hierarchical structure of top management, where leaders exerted control rather than empowering their employees. This type was more associated with work stress and fear of withholding a job [54]. However, one of the recent studies suggested that entrepreneurial leadership is an original leadership style centering on varied skills. This also includes working innovatively and creatively on shared procedures of a company to react to a vague business atmosphere and create rational strategies and get new outcomes [21].

Just like in the startup phase of new enterprises, wherein entrepreneurial leadership becomes essential, it’s critical to have robust leadership during a crisis. During a large-scale crisis, leaders are expected to navigate a volatile business environment where standard operating procedures and policies are ineffectual [55]. As a result, organizational goals are accomplished by enthusing followers to champion novelty as they become more assertive, determined and committed to strengthening and refining organizational ventures [56, 57]. Promoting employees’ confidence and self-efficacy increases the respect for the employee and empowers the entrepreneurial leadership in the company.

According to Greenberg et al. (2013), entrepreneurial leaders demonstrate a great deal of empathy [58]. They act and create opportunities to generate value for their organizations, stakeholders, and society. Entrepreneurial leaders’ task is not solely to push novel processes and goods but also to resolve business, environmental, and social issues and promote new strategic methods. Entrepreneurial leaders undertake to direct team members’ performance to achieve organizational goals, which involves exploiting and acknowledging entrepreneurial chances. Innovative and courageous entrepreneurial leaders motivate their followers with empowerment instead of rewarding or punishing them [57]. As a result of opportunity-focused behavior, employees feel better contributors to the company’s growth.

#### Job insecurity

People and companies are adversely affected by disconnections caused by the economic crisis. The extensive losing jobs can cause job insecurity, e.g., subjective perception of job uncertainty [59, 60]. Daily stressors at the workplace, specifically unexpectedly losing a job or fearing being fired will negatively affect the employer’s proactive behavior [61]. Scientists analyzed that the existence of job uncertainty decreases employees’ prosocial and proactive behaviors, where innovations are lost as a result of stressing about unemployment, instability, deficit, loss of social support, and family conflict. As an example, Bolt (2015) reported that job insecurity causes a reduction in job performance [62]. Job security is generally described as a persistent belief in employment conditions, participating in financial, social, and economic activities during the employment period in the company or a particular profession [63].

Even though there are no measurable costs of job security for employees, those employees with job security enjoy high levels of job satisfaction [64], improved work-life balance [65], and financial and psychological benefits. Employee wellbeing is regarded as a component of intellectual capital [66]. Job security represents employees’ expectations regarding the stability and longevity of their job in the organization [25]. Most employees prefer having long-term job security even when faced with significant workplace changes. Conway (2012) described forming equal exchange relationships between employers and employees [67].

Additionally, incapability to reach additional individual progress and career prospects, detrimental personal efficacy beliefs and low self-esteem, and insufficient rewards for the hard intrinsic and extrinsic work, cause a worsening in employee wellbeing and destroy professional and personal relations [68]. Also, employees who doubt job opportunities because of the crisis tend to become more worried than those who think they have other career plans [69]. Research examining the impact of experiencing failure and the psychological and behavioral implications of fear of failure was negatively related to willingness to start a new venture [70].

#### Psychological wellbeing

Psychological wellbeing is usually measured as the quality of life and overall life pleasure in the cultural context and value systems in which an individual lives [71, 72]. Self-sufficiency, control, environmental proficiency, social connection, personal efficacy and meaningful existence are considered factors of prosperity [73]. Psychological wellbeing is a construct encompassing positive affections and mental states. It refers to positive appraisals one attributes to their personal, social and workplace status, as well as those referring to the self-image [74]. The conception comprises an individual evaluation of life satisfaction in meeting the standards and goals a typical person desires to achieve [75]. As a result, individuals struggle to sustain a baseline level of happiness during various and difficult events in their lives [76, 77]. The function of work as a factor of prosperity was emphasized [78]. Employment provides circumstances for individuals to identify with society, drawing on pervasive social, economic, and governmental forces and suggesting arranged psychological efforts to alleviate the harmful effects of stressful occurrences [79]. Vital characteristics of psychological health include self-acceptance, control and autonomy, appealing to purposeful relationships and individual development [80].

## Research model and hypotheses

The conceptualized research model is empirically tested on a sample of Croatian firm employees. The variables of entrepreneurial leadership, fear of COVID-19 and job insecurity are chosen due to the immense effect they have on psychological wellbeing. Entrepreneurial leadership style is aimed towards inducing inventive and visionary spirit and paving the way to strategic planning, so employees would experience the working setting as stable, and secure, experience self-doubt less often and become resilient during adversity [21]. Followers are inspired and empowered through participation, as they become more courageous and are motivated to take risks to fight off adversities [81, 82].

According to Shuler (1980) and Cooper, Dewe and O’Driscoll (2001), not all job insecurity-related stress is bad [83, 84]. Under the proper leadership, insecurity may be transformed from stressful to a dynamic, challenge-oriented state wherein employee is faced with an opportunity to acquire new resources, skills and competencies and generate outputs that are deemed both certain and relevant [22, 23]. By engaging in problem-solving and experimentation through exploring unconventional approaches for designing a new assortment and by seeing it succeed, employees under the entrepreneurial leadership gain more confidence in their ability to navigate the erratic economic external influences and are less subject to job insecurity [24]. Furthermore, a supportive and trustworthy leader creates a safe organizational climate. When employees perceive their leader to be reliable, experienced, versatile and competent, they feel more secure about their jobs regardless of external stimuli [85]. Leadership empowerment negatively correlates with job insecurity [82]. The increase in leadership empowerment brings about a decrease in job insecurity and reduces concerns about work threats. Entrepreneurial leadership tends to decrease the perception of uncertainty during adversity due to competency, experience and expertise by linking risk and uncertainty with previous experiences [86]. When employees can rely on their leaders to make reasonable decisions, identify credible sources of income, explore novel ventures and detect new ways of addressing pressing issues, they feel less insecure about their future [87]. Entrepreneurial leadership draws from Maslow’s theory of basic human needs, thus addressing the wants for security, relatedness, autonomy and competence [88]. The more the basic needs are satisfied, the less intense the threat of job insecurity is. Entrepreneurial leaders provide their subordinates with valuable skills, knowledge and competencies, and other material and intelligible resources such as support, thus ensuring no crucial cognitive capital is lost due to an unstable organizational climate [89]. Few studies demonstrated organizational trust is key to stimulating organizational proactive behavior and improving performance [90, 91]. Entrepreneurial leaders act to reduce followers’ distress by training, boosting confidence and maintaining a positive work atmosphere [92]. Therefore, we conclude:

### Hypothesis 1: Entrepreneurial leadership has a negative impact on job insecurity

The benefits of wellbeing in the workplace are essential for employees, employers, and the country’s economy [93]. For instance, adequate levels of wellbeing in an organization would boost productivity and quality and help curb health issues leading to absenteeism [94]. Seligman and Csikszentmihalyi (2000) suggest that the connection between individuals and their working organization is formed via various processes that increase the life quality, and healthy and pleasant experiences of employees [95]. Nielsen et al. (2018) suggest that the leaders play a role in the psychological distress levels [7]. Some scientists, such as Wong (2015), require further investigation to formulate and try leadership theories for employees having informal roles [96].

To reach a high level of psychological content in the workplace, employees must be self-actualized. In other words, they should clearly understand their skills, abilities and what they can handle correctly. By investing in ensuring job security and the overall happiness of employees, businesses create a positive working environment, which ultimately increases their motivation and productivity at the workplace [85]. In addition, employees’ job security has a direct impact on health-related outcomes [2]. For example, job insecurity is strongly correlated with employee physical as well as psychological wellbeing [97].

The contextual dependence on job security suggests that every negative change in the economic and business spheres will negatively affect followers’ conception of employment. Experiencing a constant worry due to unpredictability or lack of control over one’s future, gathered with a perceived loss of intrinsic and extrinsic work benefits, is detrimental to mental welfare [98]. It leads to dysfunctional coping such as severe depression, identity disturbance, general anxiety, attention disorders and insomnia [99, 100]. Under the latent deprivation model, insecure workers feel exhausted, discouraged, dissatisfied and more likely to neglect organizational or personal development [44, 45]. Under adaptive strategies, confronting fear and insecurity entails a significant investment of resources [46]. As expected, job uncertainty increases the occurrence of mood instability, anxiety, depression and panic [47].

From this, the following is hypothesized:

### Hypothesis 2: Job insecurity has a negative effect on psychological wellbeing

Under the assumption that autonomy and control perception is fundamental to psychological wellbeing, it can be expected that COVID-19 -related instability will lead to deterioration in mental welfare [101]. The adverse effects on the human psyche have manifested in a increase in general anxiety [102], insomnia [2], depression [103] and suicidal ideation cases [70]. The concept of psychological wellbeing is closely associated with such notions as self-confidence, self-efficacy, and satisfaction. At the same time, in the workplace domain, it’s related to seizing developmental opportunities for gaining expertise, attaining professional growth and forming meaningful relationships with team leaders and coworkers. The job typically acts as an antecedent of an employee’s wellbeing, providing one with career advances and social support, such as guidance, schooling and access to extrinsic and intrinsic rewards [104]. Without the presence of significant stressors, employee wellbeing will be relatively consistent. Yet, it will be sensitive, unstable, and subject to socioeconomic changes [105].

Therefore, we hypothesize the following relationship:

### Hypothesis 3: COVID-19 has a negative effect on psychological wellbeing

Economic breakdowns are often followed by increased distress and intense uncertainty, disrupting normal business activity [106]. As unemployment becomes a significant part of everyday normalcy, panic and fear regarding career opportunities, professional advances, losing steady income, insurance, and job-related healthcare benefits sharpens [26]. COVID-19 propelled a climate of general uncertainty, with incidences of layoffs growing by the day. Stressors such as panic, anxiety, and prevailing negative emotions tend to increase negative work-related attitudes, and decrease prosocial tendencies and organizational trust, indirectly causing harmful organizational behaviors [107]. Due to its uncontrollable and adverse nature, the crisis challenges corporate performance and sustainability [108]. Employees regard leaders as responsible for ensuring their workplace stability, and for inducing trust, they should be perceived as confident, reliable, competent, trustworthy, fair, and consistent [109]. Uncertain employees are disengaged from the organizational mission, unmotivated and distressed. Coping with uncertainty is resource-consuming. Scholars define job insecurity with regard to the continued existence of the job, yet existing definitions are mostly confined to a current organization and do not include one’s overall career path or prospective occupations [110]. Therefore, when considering job insecurity, one should consider its determinants, namely, organizational and socioeconomic factors.

Job insecurity is circumstantial and varies among diverse scenarios. Considering negative changes in economic activity leading to changes in employees’ work perception, leaders should enact risk management when required. Entrepreneurial leaders emphasize the importance of teamwork, provide support and guidance to preserve followers’ wellbeing and use their influence to decrease job uncertainty [111].

Therefore, we conclude the following:

### Hypothesis 4: Fear of COVID-19 moderates the relationship between entrepreneurial leadership and job insecurity

**Fig 1:**
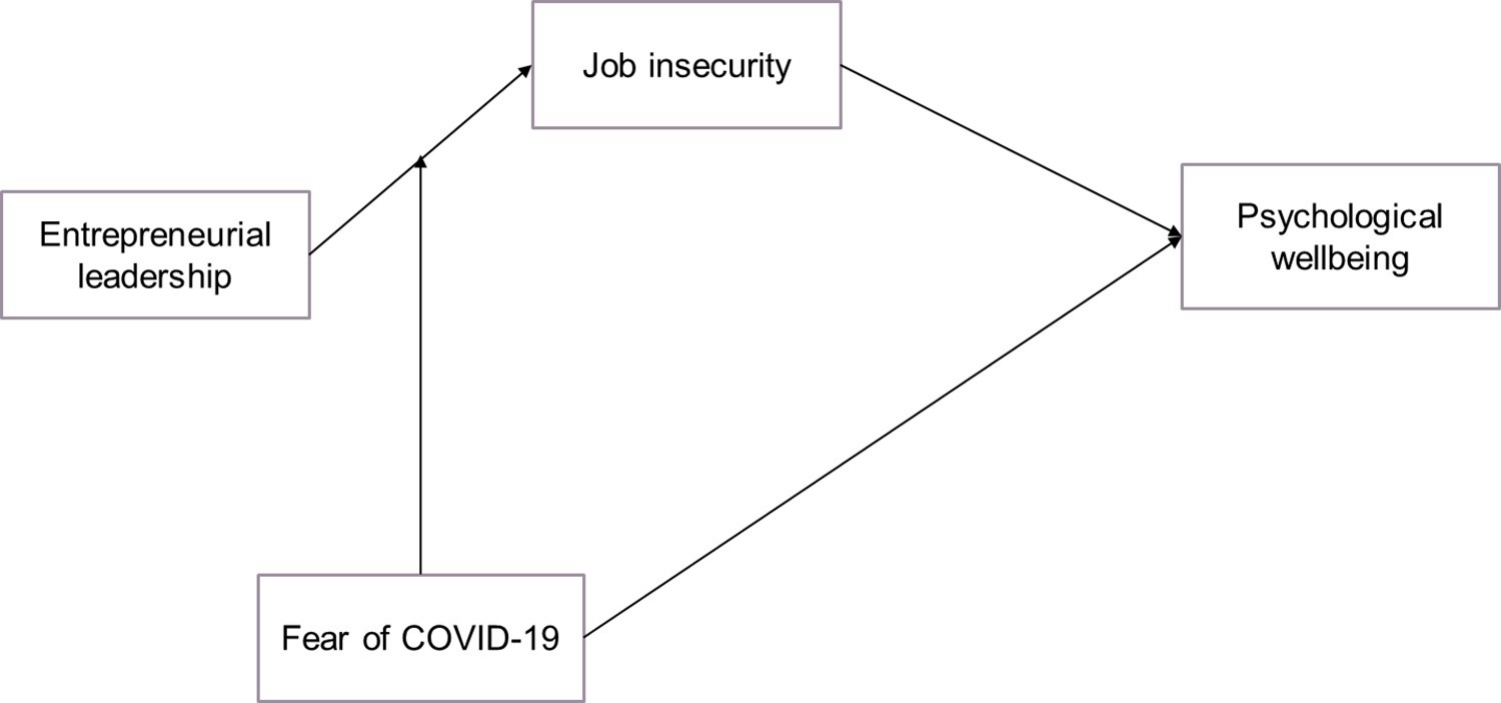
Employee leadership psychological well-being research model.

The research model of the study is presented in the Fig 1.

## Research methodology

### Research context and sample

Approval for the study was obtained verbally from the ethics committee of Wuhan University of Technology. The methods used adhere to the Declaration of Helsinki. The research study takes a deductive approach as hypotheses are formulated based on the existing theory. The study is explanatory aiming to establish a relationship between the variables. Within the test model, criteria and key variables were identified that explicitly suggest an impact on employees’ mental health in the work setting during the COVID-19 pandemic. To find out whether and to what extent they are influenced, we applied a survey strategy. A sample of 408 employees was examined. The cross-sectional survey was conducted and a questionnaire was distributed online among employees in companies and institutions located in Croatia across different industries, such as medicine, tourism, information technology and education. Demographic data, entrepreneurial leadership, fear of COVID-19, job insecurity, and psychological wellbeing data were collected via a standardized questionnaire. The survey participants received information on the investigation’s purpose before starting the questionnaire. The survey was anonymous and participants were free to leave the survey at any point in time. The data of the cross-sectional study were collected in November and December 2020, during the second wave of the COVID-19 pandemic in Croatia.

The online questionnaire comprises multi-item scales with items evaluated on a 5-point Likert scale. A total of 600 emails were sent to employees. 453 respondents accessed the survey link and completed the questionnaire. The incomplete questionnaires were not taken into consideration. After eliminating the missing values, a final sample consisting of 408 respondents was prepared for statistical analysis. Out of the 408 participants, 55 percent were female, and 45 percent were male. The majority of respondents were between ages 21 to 39 (76 percent) whereas 15 percent of respondents were between 40 and 49 years of age. The remaining respondents were 50 and older. The research sample consisted of white-collar workers with all attaining high education degrees.

### Measurements

Scales from prior studies were adopted and used to evaluate the variables of the research model. Closed-ended questions were prepared for demographic data collection. The scales used in the study exhibited a sufficient level of reliability in past studies, exceeding the Cronbach’s alpha level of 0.7. All items are measured on a five-point Likert scale, ranging from "Strongly disagree" (1) to "Strongly agree" (5), and "Very dissatisfied" (1) to "Very satisfied" (5). Due to the respondents’ high proficiency in English, the survey was conducted in the English language. The scales are presented in Table 1.

**Table 1:** Measurement scales.

Enrepreneurial leadership	The leader of this company often comes up with radically improved ideas for the products we are selling The leader of this company often comes up with ideas of completely new products that we could sell The leader of this company takes risks The leader of this company has creative solutions to problems The leader of this company demonstrates passion for his/her work The leader of this company has a vision for the future of our business The leader of this company challenges and pushes us to act in a more innovative way The leader of this company wants us to challenge the current ways we do business
Psychological wellbeing	Have you recently beenable to concentrate on what you re doing? Have you recently lost much sleep over worry? Have you recently felt you were playing a useful part in things? Have you recently felt capable of making decisions about things? Have you recently felt constantly under strain? Have you recently felt you couldn’t overcome your difficulties? Have you recently been able to enjoy your dayto-day activities? Have you recently been able to face up to your problems? Have you recently been feeling unhappy or depressed? Have you recently been losing confidence in yourself? Have you recently been thinking of yourself as a worthless person? Have you recently been feeling reasonably happy, consdering all things?
Fear of COVID-19	To what extent are you concerned about COVID-19? To what extent do you believe that COVID-19 could become a “pandemic” in your country? How likely is it that you could become infected with COVID-19? How likely is it that someone you know could become infected with COVID-19? How quickly do you believe contamination from COVID-19 is spreading in your country? To what extent has the threat of COVID-19 influenced your decisions to be around people? To what extent has the threat of COVID-19 influenced your travel plans? To what extent has the threat of COVID-19 influenced you to actually use decontamination aids (e.g. use hand sanitizer)? To what extent has the threat of COVID-19 influenced you to keep access to decontamination aids (e.g. access to hand sanitizer)?
Job insecurity	Chances are, I will soon lose my job. I am sure I can keep my job. I feel insecure about the future of my job. I think I might lose my job in the near future.

#### Psychological wellbeing

We applied Goldberg and Williams’ (1988) General Health Questionnaire-12 (GHQ-12), which is designated to evaluate individuals’ psychological wellbeing and detect disorders [112]. It gives researchers logically correct and outstanding results about the employee’s psychiatric condition. To assess the psychological wellbeing of workers in Croatia, six items have been selected. "Able to concentrate", "Capable of making decisions," and "Constantly under strain" are the items that are specifically used to estimate the psychological wellbeing of the workers.

#### Entrepreneurial leadership

A 5-point Likert scale developed by Renko et. al (2015) [57] ENTRELEAD scale consisting of eight items is used to evaluate the entrepreneurial leadership style in the company context. Sample items include: "The leader of this company often comes up with radically improved ideas for the products we are selling" and "The leader of this company takes risks". The items were evaluated from "strongly disagree" to "strongly agree."

#### Job insecurity

Job insecurity (JIS) scale was adopted from De Witte (2000) [113]. The scale evaluates personal perception on the job insecurity level among employees. Sample items include:"(1) Chances are, I will soon lose my job. (2) I am sure I can keep my job. The response options ranged from "strongly disagree" to "strongly agree" on a 5point Likert scale.

#### Fear of COVID 19

The Fear of COVID-19 was adopted in the study performed by Blakey et al. (2015), who used it to measure the fear of Ebola [114]. The Fear of COVID-19 scale was adapted and consists of 9 items measured on a 5-point Likert scale ranging from "Not at all" (1) to "Very much" (5) [114]. The scale includes sample items: " How likely is it that someone you know could become infected with COVID-19?” and “To what extent has the threat of COVID-19 influenced your decisions to be around people?”.

## Results and analysis

**Fig 2:**
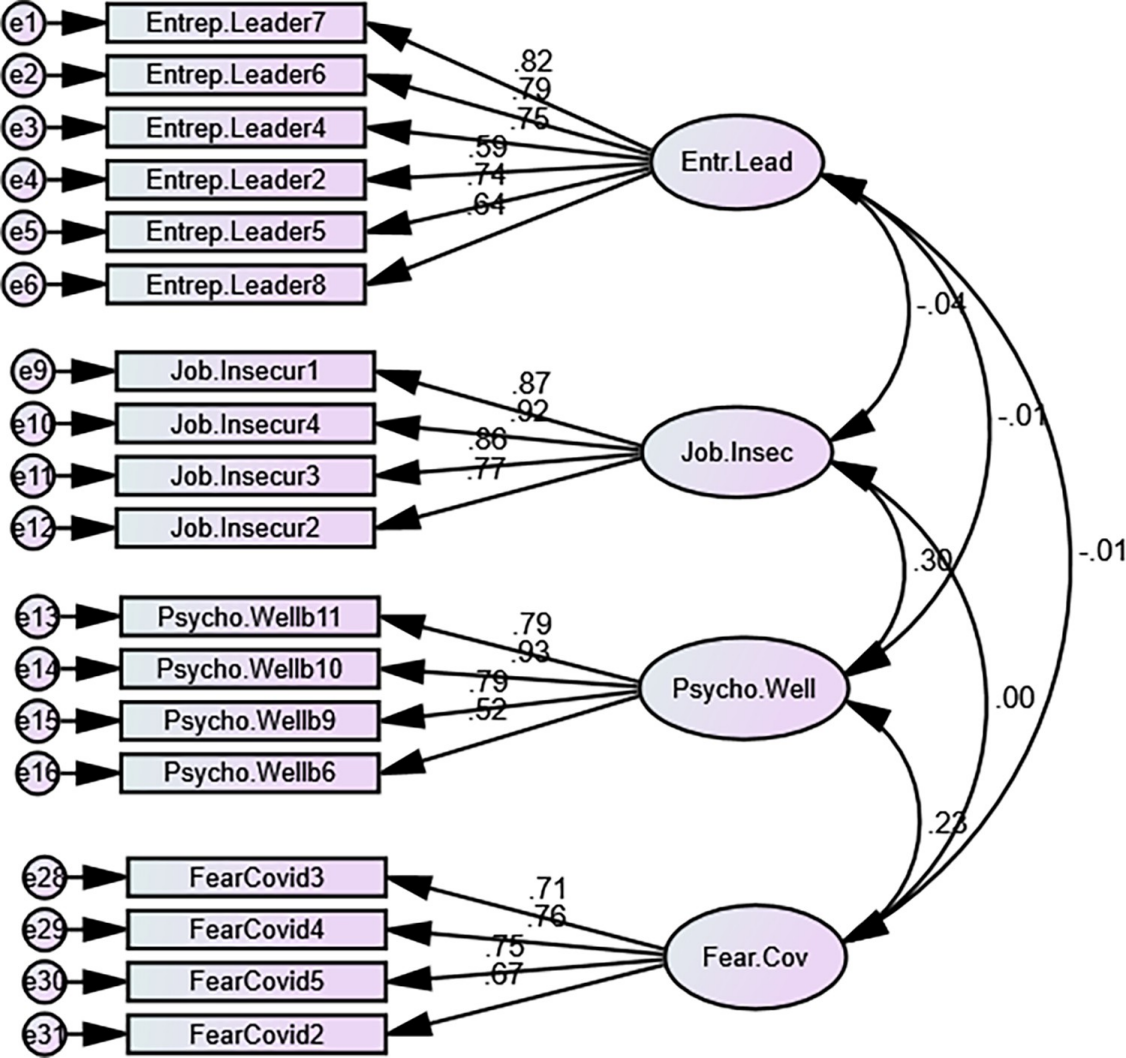
Measurement model testing.

In order to find out the answers to our research questions, we conducted a quantitative analysis using SPSS Amos 24. The missing values were eliminated, and in the end, statistical indicators for the sample of 408 respondents were analyzed. The analysis consisted of testing of the measurement tool for validity and realibility. The goodness of fit indices were computed to assess the model goodness of fit. To analyze the selected factors and validate the theoretical model, the Confirmatory factor analysis (CFA) was performed. Hypothesized relationships were tested with structural equation modeling (SEM). Path analysis was conducted and standardized parameter estimates, standard errors, and p-values were computed. All the categories of the four-factor model: Entrepreneurial leadership, Fear of COVID-19, Job insecurity, and Psychological wellbeing were examined with CFA to identify whether any alteration is required. In the measurement testing, some items were removed due to low factor loading. Both the visual measurement and standardized regression weights tables were used to identify the elements for removal. The CFA analysis is presented in Fig 2. All indexes verified good model fit except for CFI and RMSEA (χ2/df = 4.062; CFI = 0.779; SRMR = 0.087; RMSEA = 0.087). Bollen–Stine p<0.05 indicated poor fit. Standardized regression weights analysis also indicated several items with low loading factors. Convergent Validity was calculated indicating that the AVE for Entrepreneurial Leadership, Psychological wellbeing and Fear of Covid is less than 0.50. Results are displayed in Table 2. To improve validity and consequently model fit, items with low factor loading were removed.

**Table 2:** Model validity measures.

Variable	CR	AVE	MSV	MaxR(H)	Entr.Lead	Job.Insec	Fear.Cov	Psycho.Well
**Entr.Lead**	0.878	0.477	0.005	0.89	**0.69**			
**Job.Insec**	0.916	0.734	0.11	0.927	0.017	**0.856**		
**Fear.Cov**	0.867	0.423	0.041	0.876	0.069	0.025	**0.651**	
**Psycho.Well**	0.762	0.286	0.11	0.894	0.034	0.332***	0.203***	**0.535**

Removal of low loading factors improved CFI up to 0.947, together with Bollen–Stine p>0.05. All other indicators illustrated excellent fit of the model (χ2/df = 2.52; SRMR = 0.052; RMSEA = 0.061). The goodness of fit indices are displayed in Table 4. A convergent validity check was performed through the average variance extracted (AVE) value. Convergent validity can be confirmed with construct reliability (CR) only. Based on that, all the variables have a good CR value of above 0.7, and namely Entrepreneurial leadership (CR = 0.868), Job insecurity (CR = 0.916), Fear of COVID-19 (CR = 0.814) and Psychological Wellbeing (CR = 0.851). Discriminate validity presented by Maximum shared variance (MSV) also shows acceptable validity for all the variables. CFA has performed again. After deleting items due to bad validity and model fit satisfactory levels were achieved. The results are displayed in Table 3.

**Table 3:** Model validity measures.

Variable	CR	AVE	MSV	MaxR(H)	Entr.Lead	Job.Insec	Fear.Cov	Psycho.Well
**Entr.Lead**	0.868	0.526	0.002	0.88	**0.725**			
**Job.Insec**	0.916	0.733	0.088	0.927	0.04	**0.856**		
**Fear.Cov**	0.814	0.524	0.054	0.818	0.011	0.005	**0.724**	
**Psycho.Well**	0.851	0.598	0.088	0.911	0.006	0.296***	0.233***	**0.773**

All indexes verified good model fit except for CFI (χ2/df = 2.699; CFI = 0.741; SRMR = 0.075; RMSEA = 0.078). Bollen–Stine p<0.05 indicated poor fit. Removal of low loading factors with an estimated value below 0.04 and above 0.04 improved CFI up to 0.769. However, still indicating poor marginal fit together with Bollen–Stine p<0.05.

Modification of indices was performed by correlating high within-item errors in Job insecurity and psychological wellbeing. Removal of items with residual covariance of more than two resulted in significant improvement Bollen–Stine p>0.05 and CFI, where the confirmatory factor index reached an acceptable level (CFI = 0.901). As other indicators illustrated excellent fit of the model (χ2/df = 1.802; SRMR = 0.057; RMSEA = 0.054). A convergent validity check was performed through the average variance extracted (AVE) value. Convergent validity can be confirmed with construct reliability (CR) only. Based on that, almost all the variables have a good CR value of above 0.7, namely Entrepreneurial leadership (CR = 0.784), Job insecurity (CR = 0.877) and Fear of COVID-19 (CR = 0.768). Discriminate validity presented by Maximum shared variance (MSV) also shows acceptable validity for them. CR for Psychological wellbeing is almost reaching a value of 0.7.

The structural model has been tested. In addition, the model fit measures were calculated. As the first step, though, the relationships were examined in CFA to verify good model fit. The obtained results indicate good model fit (χ2/df = 2.482; CFI = 0.948; SRMR = 0.052; RMSEA = 0.06). The model fit measures are displayed in Tables 4 and 5.

**Table 4:** Model fit measures.

Measure	Estimate	Threshold	Interpretation
CMIN	325.105		
DF	129		
CMIN/DF	2.52	Between 1 and 3	Excellent
CFI	0.947	>0.95	Acceptable
SRMR	0.052	<0.08	Excellent
RMSEA	0.061	<0.06	Acceptable
Pclose	0.014	>0.05	Acceptable

**Table 5:** Model fit measures of the structural model.

Measure	Estimate	Threshold	Interpretation
CMIN	325.142		
DF	131		
CMIN/DF	2.482	Between 1 and 3	Excellent
CFI	0.948	>0.95	Acceptable
SRMR	0.052	<0.08	Excellent
RMSEA	0.06	<0.06	Acceptable
Pclose	0.02	>0.05	Acceptable

Hypothesized relationships were tested with structural equation modeling (SEM). The structural model with a direct relationship between Entrepreneurial leadership, Job insecurity, and Fear of COVID-19 and Psychological wellbeing showed an excellent fit with no need for further modifications (χ2/df = 1.945; CFI = 0.901; SRMR = 0.059; RMSEA = 0.059). Thus, for every increase in the raw score unit of Entrepreneurial leadership, there is an increase of 0.008 for the raw score unit in Psychological wellbeing (β = 0.007). Even though the relationships are in line with the theoretical assumptions, the p-value is higher than 0.05 resulting in acceptance of null hypotheses. The results of the path analysis are displayed in Tables 6 and 7.

**Table 6:** Moderation regression weights.

Dependent variable	Path	Independent variable	Estimate	SE.	CR.	P
ZJob.Insec	<	ZEntr.Lead	0.045	0.05	0.902	0.367
ZJob.Insec	<	ZEntr.Lead_x_ZfearCov	0.022	0.041	0.532	0.595
ZPsycho.Well	<	ZEntr.Lead	0.009	0.045	0.196	0.845
ZPsycho.Well	<	ZFear.Cov	0.265	0.045	5.855	***
ZPsycho.Well	<	ZEntr.Lead_x_ZfearCov	0.021	0.038	0.553	0.58
ZPsycho.Well	<	ZJob.Insec	0.318	0.045	7.04	***

Significance level ***p <0.001

**Fig 3:**
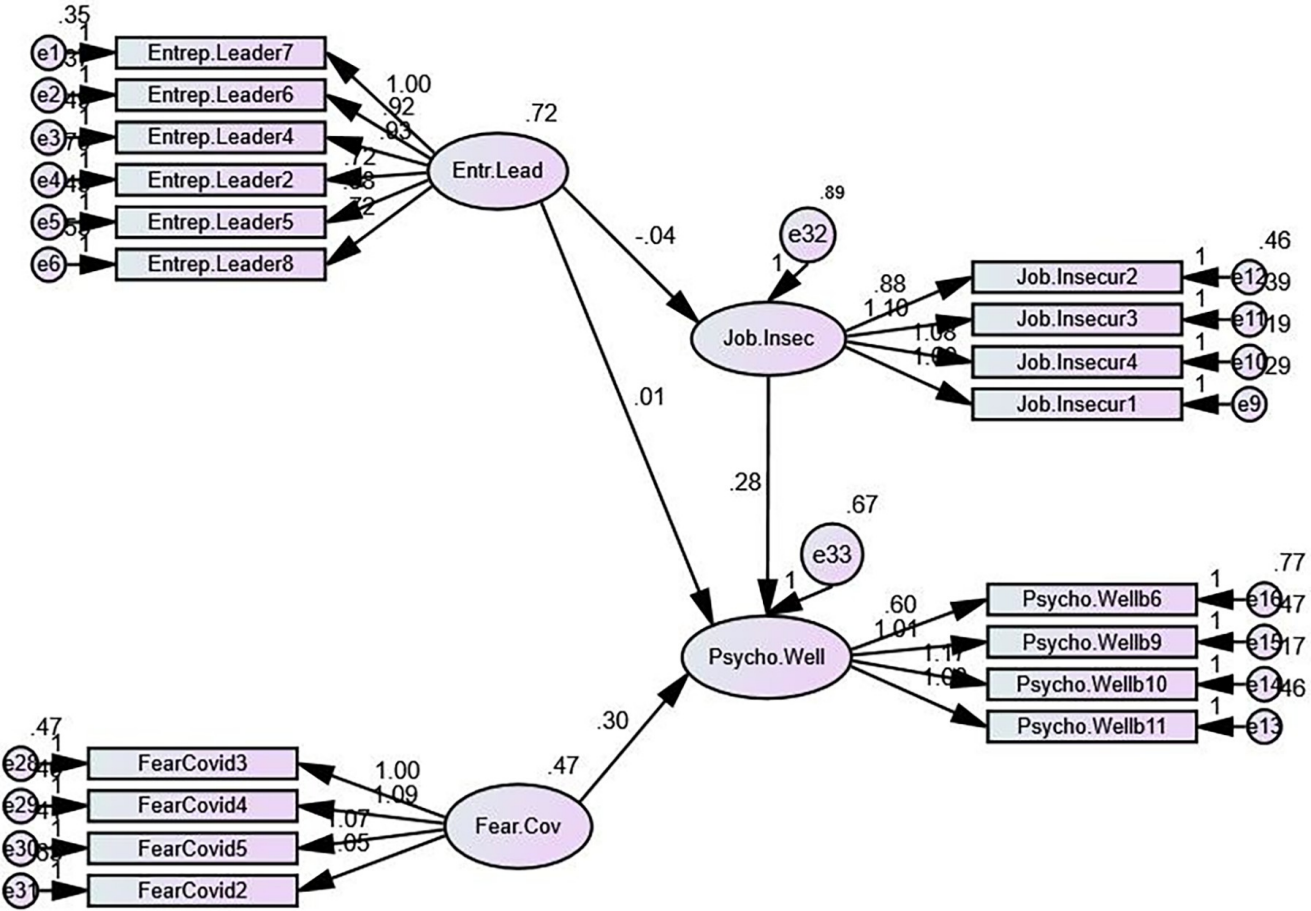
Path analysis and confirmatory factor analysis.

There is a decrease of 0.044 in the raw score unit for Job insecurity with Entrepreneurial leadership (β = 0.04). As for Job insecurity negatively predicting Psychological wellbeing, a direct connection between them was confirmed, the raw coefficient is equal to 0.277 with β = 0.296, with the p-value reaching the desired level of significance. To sum up, hypothesis H1 stating that Entrepreneurial leadership impacts job insecurity has been rejected. On the other hand, the H2 stating that Job insecurity has a positive effect on psychological wellbeing was confirmed, as well as H3 stating that Fear of COVID-19 has an impact on psychological wellbeing. The structural model is displayed in Fig 3.

**Table 7:** Path analysis parameters, errors and p-values.

Dependent variable	path	Independent variable	Standardized weights	Estimate	SE.	CR.	P
Job.Insec	<	Entr.Lead	0.04	0.044	0.061	0.726	0.468
Psycho.Well	<	Entr.Lead	0.007	0.008	0.054	0.141	0.888
Psycho.Well	<	Job.Insec	0.296	0.277	0.049	5.604	***
Psycho.Well	<	Fear.Cov	0.232	0.299	0.073	4.123	***
Entrep.Leader7	<	Enter.Lead	0.818	1			
Entrep.Leader6	<	Entr.Lead	0.786	0.917	0.054	16.993	***
Entrep.Leader4	<	Entr.Lead	0.746	0.926	0.058	15.947	***
Entrep.Leader2	<	Entr.Lead	0.59	0.722	0.06	12.044	***
Entrep.Leader5	<	Entr.Lead	0.744	0.884	0.056	15.902	***
Entrep.Leader8	<	Entr.Lead	0.64	0.716	0.054	13.246	***
Job.Insecur1	<	Job.Insec	0,868	1			
Job.Insecur4	<	Job.Insec	0.919	1.084	0.042	25.498	***
Job.Insecur3	<	Job.Insec	0.858	1.103	0.048	22.811	***
Job.Insecur2	<	Job.Insec	0.774	0.877	0.046	19.145	***
Psycho.Wellb6	<	Psycho.Well	0.517	0.601	0.057	10.488	***
Psycho.Wellb9	<	Psycho.Well	0.794	1.01	0.058	17.391	***
Psycho.Wellb10	<	Psycho.Well	0.93	1.172	0.06	19.605	***
Psycho.Wellb11	<	Psycho.Well	0.793	1			
FearCovid2	<	Fear.Cov	0.671	1.048	0.091	11.562	***
FearCovid5	<	Fear.Cov	0.752	1.071	0.085	12.609	***
FearCovid4	<	Fear.Cov	0.763	1.093	0.086	12.711	***
FearCovid3	<	Fear.Cov	0.705	1			

Significance level ***p <0.001

In the process of SEM testing, we assumed that Fear of COVID-19 moderates the relationship between entrepreneurial leadership and job insecurity. Such effects were not found, but there was a significant impact of Fear of COVID-19 on psychological wellbeing, thus accepting Hypothesis 3 and rejecting Hypothesis 4. Also, additional analysis showed that Job Insecurity is not a mediator of the relationship between Enter. Leader and Psycho. Well.

## Discussion

The findings of the current study show that job security is one of the significant factors which contribute positively to ensuring mental health. It is essential to mention that over the past years the attention of occupational mental health has increased. Therefore, understanding the main factors and causes of mental health is essential to prevent future incidents. Although there is much discussion about the exact definition of the psychological wellbeing concept, its significance in the workplace has never been debated. Entrepreneurial leadership is still an under-explored leadership style and should be beneficial for guiding and influencing the performance of team members to achieve organizational goals. Lack of support for the hypothesized effect can be due to major leadership focus on the exploitation of entrepreneurial opportunities and lesser direct attention to employees’ economic and health preoccupations, such as distress and social strain. However, certain leadership qualities should be acknowledged as conducive to developing a sense of autonomy and eventually decreasing the overall fear and insecurity.

Findings of the study show that job insecurity and fear of COVID-19 are critical factors of employee wellbeing. With a detailed description of the situation in the studied sample, the value of each factor for psychological wellbeing has been determined, and from the results, we derived a recommendation for attaining higher levels of psychological welfare.

We found the positive impact of entrepreneurial leadership on job security was insignificant, therefore rejecting hypothesis 1. Our study could not corroborate the results of previous studies arguing that entrepreneurial leadership tends to decrease the perception of uncertainty during adversity due to competency, experience, and expertise by linking risk and uncertainty with previous experiences [86]. Our results failed to corroborate the conclusions of Tafvelin et.al, (2018) [115].

Hypothesis 2 stating job insecurity harms psychological wellbeing was accepted. Our findings are in line with the findings of Prawitz et al. (2006) [116], Disney and Gathergood (2013) and Godinic et al. (2020) [78, 117]. A job typically acts as an antecedent of an employee’s wellbeing, providing one with career advances and social support, such as guidance, schooling and access to the extrinsic and intrinsic reward. Without the presence of significant stressors, employee wellbeing is relatively consistent. However, the opposite is true when participants are subject to socioeconomic changes [105]. As unemployment increased over the last two years due to the pandemic, panic and fear regarding career opportunities, professional advances, losing steady income, insurance, and job-related healthcare benefits sharpened.

Hypothesis 3 stating that the fear of COVID-19 negatively impacts psychological wellbeing is accepted. This is consistent with the findings of Casale and Flett (2020) [118], Aguiar Quintana et al. (2021), Obrenovic et al. (2020) [2], Koçak and Younis (2021) [119] and Di Blasi et al. (2021) [120]. In relation to the previous hypothesis, we find that in the work setting, the COVID-19 pandemic severely undermines self-confidence, efficiency, productivity, work enthusiasm and the capability to involve in work actively. The pandemic which has caused insecurity in the workplace interferes with all fundamental dimensions of wellbeing. Stressors such as panic, anxiety, and prevailing negative emotions tend to increase negative work-related attitudes, and decrease prosocial tendencies and organizational trust, indirectly causing harmful organizational behaviors [108]. Our findings are in line with the work of Yu, Park and Hyun (2021) and Henokh Parmenas (2021) [121, 122].

Hypothesis 4 stating there is a moderating effect of EL on the relationship between job insecurity and fear of COVID-19 was rejected. There are several possible explanations for this outcome. It may be that the main concern of entrepreneurial leaders is related to organizational achievement, novelty, sustaining liquidity and activity. However, under the proper leadership employees can build resilience towards uncertainty by ensuring the additional resources, such as knowledge, skill, experience, and entrepreneurial disposition and thus begin to focus less on adversity and more on advancement opportunities. A reasonable explanation of the result turning insignificant is due to the multidimensional nature of entrepreneurial leadership, and all components of the construct not needing to have the same impact. Further investigation is warranted to confirm the hypothesis. Future studies are advised to single out specific aspects of EL, such as empowerment, and look into their effect on job security and psychological wellbeing.

Notwithstanding the lack of agreement, we believe our findings compare well with the latest developments in mental health and occupational psychology during COVID-19.

### Theoretical implications

This study is the first step toward enhancing our understanding of the entrepreneurial dimension of transactional psychology. The observations we recorded have implications for research into the study of mental processes and their impact on organizational proactive behavior. We provide the previously overlooked concept of EL with additional insight on how to create a healthy and people-centric organizational climate during market uncertainty and complexity. The evidence from this study points toward the idea that through empowerment and positive stimulus, which is aimed at affecting self-efficacy beliefs and self-determination, employees will feel more in charge, secure and balanced. The strong point of our contribution lies in the finding that the entrepreneurial leadership style alone does not buffer against job insecurity, thus pointing that a more comprehensive inquiry into empowering aspects should be carried out in the future to find out which incentives leaders can use to boost employees’ confidence. Taken together, our findings implicate that job insecurity stems from more than leadership and includes other organizational factors, such as coping, learning abilities, developmental opportunities, personal disposition, and pressure bearing. Therefore, mere leadership is not likely to significantly reduce the uncertainty when isolated from related influential aspects. Returning to the question posed at the beginning of the study, it is now possible to state that although we didn’t manage to generate firm evidence of EL’s moderation effect on the relationship between job security and psychological health, we still contend that solid leadership should be implemented during critical times. In our view, these results represent an excellent initial step toward identifying steps to achieve greater psychological wellbeing in the workplace. Our research suggests that the policy makers should encourage flexibility, creativity, posing questions and problem-solving to increase employees’ autonomy. Furthermore, it is crucial to minimize fear-based rigidity and provide opportunities for employees to adapt, improve and experiment.

### Limitations of the study

The first limitation is that since data is cross-sectional, it only makes results applicable and relevant for the given time frame during which the given study was conducted. Thus, it is also essential to conduct longitudinal research to assess psychological wellbeing and other factors. Furthermore, this study focused on employees in Croatian companies, without choosing a particular industry.

Next, the data collection relied on self-reported measures. There is always a risk that the respondents did not reply honestly; however, due to the anonymous nature of the questionnaire, the threat has been minimized. Furthermore, the respondents were advised to reply truthfully and objectively before starting the questionnaire. To render research findings more reliable and accurate, it would be desirable to consider observations of organizational data measuring job losses, employee turnover, etc. Job insecurity is not impacted by entrepreneurial leadership. Job security may not only arise from organizational practices or leadership but also from individual capacity, and self-efficacy. Theoretically, the relationships are supported, but our study was not able to prove that the relationships are statistically significant in the selected research sample. Thus, the study should be repeated in different contexts, potentially selecting a more homogenous sample, to validate the hypothesized relationships. It is also important to replicate and conduct similar studies on the same topic due to the changing environment and adjustment to the COVID-19 pandemic.

### Future studies

Additional research can be conducted to understand the other psychosocial factors that are essential for healthy work environments. Although mental health is a concern for both workers and organizations, problems are usually unpredictable, and methods for better human resource management and mental health increases should be developed. Other variables such as burnout and stress, organizational practices, work-family conflict and many others could be introduced in the model. Job security may not only stem from leadership or other organizational factors but also from people’s capacity, and self-efficacy, so more research on job security during the COVID-19 pandemic is warranted. Future studies could therefore calculate the value of all additional factors. This will provide more insight into the coping of companies and employees during the COVID-19 pandemic. The mechanism of psychological wellbeing could also be explained using some other grounded theory, such as Maslow’s Theory of needs. Future research should aim to compute the predictive value of all new variables. In that way, a more detailed description of the situation of the COVID-19 pandemic business implications on more varied samples of employees will be ensured. Therefore, those potential variables warrant more investigation. As entrepreneurial leadership did not have a significant impact, and as leaders play a role in the psychological distress levels of employees it is important to further investigate the topic [7]. Future studies could have a more precise focus on particular types of companies or industries. Therefore, the same research model could be validated in the context of a different, more homogenous sample.

## Conclusion

The present study investigated the number of different factors that contribute to psychological wellbeing in organizations. Particularly, these factors include entrepreneurial leadership, fear of COVID-19 and job security. Psychological wellbeing is considered one of the critical elements of the organization’s corporate policy. Since employees are an essential part of every organization, ensuring their psychological safety should be one of the main priorities of every organization. Most organizations lack the proper understanding of mental health, thus, it leads to a number of cases that harm the accomplishment of the work and at the same time, there are adverse effects on the company’s performance. The novelty of the current research can be seen in the fact that it has proposed a certain number of novel factors such as job insecurity, and fear of COVID-19. Job security and managing fear of COVID-19 are critically important not only for ensuring the proper management of employees but also for ensuring their mental health. The findings of the current study show that a higher priority should be given to ensuring organizations’ welfare and sustaining employees’ psychological wellbeing, alongside their other important goals such as financial, marketing and operations. This implies that the mental health of employees should be given the same level of attention as the company’s other main goals. Another important contribution of the current research can be seen from the employee’s perspective. In other words, employees should also be educated about the importance of psychological wellbeing and its implications of it for their job performance, and overall organizational success. In other words, they should be given relevant information about the existence of such mental states and ways of dealing with adversity during the COVID-19 pandemic. Employees should be provided with enough training regarding the job they do and psychological impacts, as well as coping with difficult environments and outside risks, such as the COVID-19 pandemic. This allows them to prevent the negative consequences related to psychological distress. We posit that the leadership variable bears even more significance to organizational sustainability by sustaining employees’ welfare in unstable circumstances than during stability. We call upon further investigation on the influence of specific leadership styles, as well as their inherent characteristics on mitigating the psychological distress related to job insecurity.

## Supporting information

S1 Data (XLSX)
